# Age-related differences in the attentional white bear

**DOI:** 10.3758/s13423-019-01622-9

**Published:** 2019-06-10

**Authors:** Brandon K. Ashinoff, Yehoshua Tsal, Carmel Mevorach

**Affiliations:** 1 School of Psychology, University of Birmingham, Edgbaston, UK; 2 Centre for Human Brain Health, University of Birmingham, Edgbaston, UK; 3 Department of Psychiatry, Columbia University, New York, NY USA; 4 School of Psychological Sciences, Tel Aviv University, Tel Aviv, Israel; 5 Department of Behavioral Sciences, Zefat Academic College, Safed, Israel

**Keywords:** Aging and attention, Cognitive aging, Cognitive and attentional control, Cognition and aging

## Abstract

The cognitive aging literature suggests that aging populations exhibit impairments in the proactive inhibition of attention. Although proactive inhibition is often preceded by the allocation of attention toward the predicted or known spatial location of to-be-ignored stimuli, proactive allocation of attention has not been assessed in aging populations. In this study, an older and younger cohort engaged in the attentional-white-bear paradigm which measures proactive allocation of attention. In this task, on 80% of trials, participants must identify a centrally located letter surrounded by congruent or incongruent flanker letters. The flanker locations are fixed and predictable within each block of the study. On 20% of trials, they must identify which of two dots appear first on the screen. One dot appears in the same location as the flanker, and one appears in an empty location during the flanker task. The typical white-bear effect is that, despite the dots appearing at the same time, participants more often report the dot in the location of the flanker (i.e., the potentially to-be-ignored location) to appear first. The magnitude of this effect is interpreted as the magnitude of attentional allocation prior to inhibition. In Experiment 1, there was no difference in the magnitude of the attentional white bear between younger and aging cohorts. However, when the attentional system was sufficiently taxed by reducing the flanker presentation (Experiments 2a and 2b), age-related differences emerged. In particular, older participants showed a reduced white-bear effect, reflecting a potential impairment in the proactive allocation of attention toward the location of expected distractors.

As we age, many changes take place that alter our cognitive abilities (Craik & Salthouse, 2011; Grady, 2012; Hedden & Gabrieli, 2004; Persson et al., 2006). The dual-mechanisms theory of proactive and reactive control (DMC; Braver, 2012) argues that cognitive control is driven by two primary mechanisms. Proactive control is an “early selection” mechanism that allows one to select and maintain goal-relevant information prior to stimulus presentation in order to prepare a response to a given stimulus based on prior knowledge. Reactive control is a “late correction” mechanism that allows one to alter behavioral plans in the moment when suddenly presented with new and relevant information. Recent research has shown that older participants tend to favor reactive over proactive control strategies (Jimura & Braver, 2010; Paxton, Barch, Racine, & Braver, 2008), due to a presumed age-related impairment to proactive control mechanisms.

These studies generally report age-related impairments related to the proactive *inhibition* of attention. This is important because several researchers have argued that a prominent aspect of cognitive aging is a decline in the ability to proactively ignore (i.e., inhibit) distracting information (the inhibition-deficit theory of cognitive aging; Braver, 2012; Hasher, Stoltzfus, Zacks, & Rypma, 1991; Hasher & Zacks, 1988; Kramer, Humphrey, Larish, & Logan, 1994; Lustig & Jantz, 2015). However, some accounts of inhibition processes suggest that an initial allocation of attention toward distractors is necessary for their (ostensibly proactive) inhibition. This may have important implications for inhibition-deficit theories of cognitive aging (e.g., Lustig, Hasher, & Zacks, 2007) because impairments in attentional allocation prior to inhibition could result in cascading effects that would appear as inhibition deficits. Essentially, it may be more difficult to inhibit something if you are bad at identifying and locating it in the first place. To date, few studies have attempted to assess proactive *allocation* of attention in aging populations. This study attempts to fill in this gap in the literature by investigating whether proactive allocation of attention prior to inhibition is impaired in aging populations.

One method that has been used to measure proactive allocation prior to inhibition is the preview search paradigm (Watson & Humphreys, 1997), in which participants have to search for a predefined target among distractors, but a subset of the distractors is presented (the preview display) at least 400 ms prior to the target display. Typically, it is found that participants are able to inhibit the preview array, so that search is restricted to the new items presented later (as if the new items are presented on their own). Interestingly, using dot probes (Allen, Humphreys, & Matthews, 2008; Humphreys, Stalmann, & Olivers, 2004; Olivers, Humphreys, & Braithwaite, 2006) and electrophysiological measures (Belopolsky, Peterson, & Kramer, 2005), it has been demonstrated that the relevant to-be-ignored locations are initially proactively attended to and identified prior to adopting an inhibitory bias against the locations of the previewed distractors—a process called visual marking. Using the preview search paradigm with older participants, Watson and Maylor (2002; Kramer & Atchley, 2000; Kramer & Kray, 2006) showed that older participants produced standard visual marking effects, as long as static displays were used. While this points to a possible difference in how older participants search static versus dynamic displays (also see Becic, Kramer, & Boot, 2007), it is unclear if this is related to the initial allocation of attention to the preview items or to differences in inhibition processes. Therefore, while this may suggest that proactively allocating attention toward to-be ignored distractors is intact in old age, it is by no means conclusive.

Other paradigms have also been used to assess proactive allocation of attention prior to inhibition in young, neurotypical cohorts. For example, Moher and Egeth (2012; Cepeda, Cave, Bichot, & Kim, 1998; Cunningham & Egeth, 2016; Jollie, Ivanoff, Webb, & Jamieson, 2016; Munneke, Van der Stigchel, & Theeuwes, 2008) showed that cueing nontarget (i.e., distractor) features (i.e., IGNORE RED) resulted in the allocation of attention toward a to-be-ignored item prior to inhibition in a visual search task (also measured using detection of a probe dot). In another example, Max and Tsal (2015) characterized the temporal dynamics of proactive allocation and inhibition. Participants were presented with a flanker task that began with identical target and distractor items. However, at a random interval during the trial, the distractor items would mutate into incongruent or neutral distractors. They found that performance was impaired if the distractor mutation occurred within the first 50 ms of stimulus presentation, suggesting that at least some attention was allocated toward them early on. If the mutation occurred after 50 ms, the new identities of the flanker items were successfully inhibited and had no effect on task performance. However, Max and Tsal (2015) noted that in their paradigm the process they characterized might be preattentive or reflective of an attentional “zoom lens” contracting around the target, rather than a shift from allocation to inhibition. Crucially, none of these paradigms were tested with an older cohort, and it is therefore still not clear whether age-related differences in proactive attentional allocation occur.

A more direct measure of proactive allocation of attention to distractors is provided in the attentional-white-bear (AWB) paradigm (Tsal & Makovski, 2006). In the AWB paradigm, participants engage in a primary flanker task—identifying a central letter flanked by two diagonal distractors (e.g., top left and bottom right) appearing in the same locations throughout a block of trials. However, on a small minority of trials (20%), a temporal-order-judgment task appears instead of the flankers’ display, in which participants indicate which of two simultaneously presented dots appeared first. The purpose of the flanker task is to induce an expectation that participants will have to ignore a flanker at a specific location and time (the flanker location was blocked, and the inter-stimulus interval (ISI) was consistent throughout the study). The flanker appears in the same location as one of the two dots, so the participants are preparing to inhibit a flanker, but get a dot instead on some trials. The critical finding (Tsal & Makovski, 2006) is a tendency to identify the dot that appeared at an expected distractor position as appearing before the dot occupying the expected empty location—referred to as the attentional-white-bear effect. In fact, the effect holds even when significant perceptual, memory, and sensory constraints are placed on the flanker task (Lahav, Makovski, & Tsal, 2012). This bias toward the expected (to-be-ignored) flanker position is attributed to attention allocation to the expected flanker position prior to the flanker presentation and therefore represents a direct measure of proactive allocation of attention to distractors prior to expected inhibition. Thus, the magnitude of the AWB effect is a metric representing the proactive allocation of attention.

It should be noted that we are not claiming that all of these paradigms are necessarily reflective of the exact same process. However, each of these paradigms arguably engage the proactive allocation of attention ostensibly as a precursor to the proactive inhibition of attention. Crucially, these processes do not necessarily engage proactive allocation and inhibition in the exact same manner, leading to distinct inhibition processes based on similar mechanisms. In other words, even if the attentional-white-bear, visual marking, and other studies do not reflect the exact same process, they do engage the same mechanism (proactive allocation of attention) as part of those processes, and our test of this mechanism has implication for all of the processes in which it is engaged.

To directly assess proactive allocation of attention to distractors in aging populations, we employed the fixed-block attentional-white-bear paradigm (Tsal & Makovski, 2006) in two groups of younger and older healthy adults. Specifically, we ask whether the magnitude of the white-bear effect (proportion of responses identifying the dot appearing in the expected distractor location as appearing first in time, relative to those appearing at the expected empty location), which arguably reflects the magnitude of the resources proactively allocated to the expected distractor location, differs between younger and older participants. In addition, we also manipulated the contrast of the flankers to include both equal-contrast (compared with the target) and low-contrast flankers. Low-contrast flankers may require more attention to be allocated to them within an allocate-first scenario, and this enables us to test whether differences in the AWB effect between the age groups emerge when flankers are harder to process. Thus, two versions of the task were developed. In one, the flanker distractors are equal in contrast to the target letters. In the second version, the flanker distractors are of significantly lower contrast than the target letters.

## Experiment 1: Methods

### Power analysis

An a priori statistical power analysis for a repeated-measures ANOVA was performed for sample size estimation, based on data from Lahav et al. (2012, Experiment 1). In this study, they found a significant white-bear effect, defined as a main effect of distractor location in a temporal-order-judgment task—similar to the one used in our study. Although not reported in the original paper, through personal communication with the authors we obtained the partial eta squared (${\upeta}_{\mathrm{p}}^2$= .242) of the white-bear effect in this study, which converted to an effect size *f* (U) of 0.565. (The other parameters of the analysis were set as the following: alpha = .05, power = 0.95, number of groups = 2, number of measurements = 2, nonsphericity correction = 1.) Based on this, the total projected sample size (across all groups) needed to detect a white-bear effect with an effect size of .565 (G*Power, Version 3.1.9.2; Faul, Erdfelder, Buchner, & Lang, 2009; Faul, Erdfelder, Lang, & Buchner, 2007; Lakens, 2013, Appendix A) is 46 for a repeated-measures ANOVA with a within–between subjects interaction. Thus, an ideal study would include 23 participants per group to have a power of .956 (see Appendix A). In Experiment 1, there are 21 younger and 22 older participants (*N* = 43; a priori power = .942; see Appendix A). In Experiment 2a, there are 20 younger and 20 older participants (*N* = 40; a priori power = .924; see Appendix A). In Experiment 2b, there are 20 younger and 19 older participants (*N* = 39; a priori power = .917; see Appendix A).

### Participants

Twenty-five younger and 26 older participants took part in this study; however, because of technical issues during a couple of experiment sessions, we had to exclude two younger and two older participants. We further excluded two younger and two older participants because of poor performance (>20% incorrect button presses during the temporal-order-judgment task). Twenty-one younger (*M*_age_ = 18.71 years, *SEM*_age_ = 0.18, age range: 18–21 years, 20 females) and 22 older (*M*_age_ = 70.77 years, *SEM*_age_ = 1.37, age range: 60–82 years, 13 females) participants were included in the final analysis. The two groups participated in two successive behavioral experiments. The order of the tasks was counterbalanced to account for possible fatigue and order effects. Younger participants were recruited from the undergraduate population in the school of psychology at the University of Birmingham, UK. They were compensated for their participation with course credits. The older participants were recruited from a volunteer pool maintained by the School of Psychology at the University of Birmingham. They were compensated for 1.5 hours of their time with a one-time payment of £7. All participants had to sign an informed consent form prior to the study. Participants were healthy with no history of head injury, mental health issues, or neurological disorders. The older participants were screened for decline in cognitive functions using the Montreal Cognitive Assessment (MoCA). All of the older participants scored within the normal range (*M*_score_ = 27.5, *SEM*_score_ = .23).

#### Stimuli and procedure

**Fig. 1:**
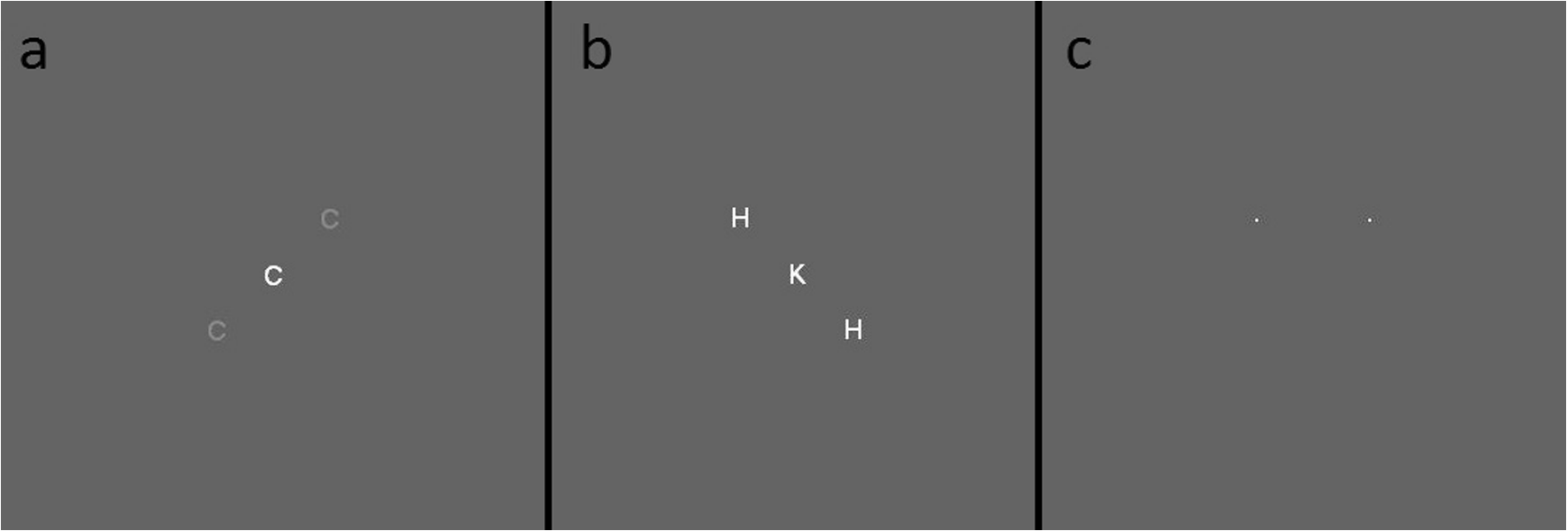
**a** The low-contrast display with distractors in the upper right/lower left flanker configuration. **b** The equal-contrast display with distractors in the upper left/lower right flanker configuration. **c** The dot display

Two versions of the attentional-white-bear task (Lahav et al., 2012; Tsal & Makovski, 2006) were used. These versions were the same except where indicated below. Color was defined using RGB color coordinates. The background color of the screen thorough the experiment was gray [100, 100, 100]. Participants were presented with four blocks of 180 trials each. Each block consisted of 80% (144) flanker displays (see Fig. 1a–b) and 20% (36) two-dot displays trials (see Fig1 c). These displays were randomly intermixed with the exception that two-dot displays could not appear consecutively. Flanker trials consisted of three letters oriented along a diagonal through the center of the screen. Upon being presented with a flanker display, participants had to identify the central letter and respond based on its identity. The central letter was randomly drawn from *H*, *K*, *C*, or *S*. Participants were required to press the *A* key if the central letter was an *H* or a *K*, and the *L* key if the central letter was a *C* or an *S*. The two flanking letters were also drawn from the same group of four letters, though both distractor letters were always the same within a trial. Therefore, on each trial the distractor letter could be congruent or incongruent with the correct response to the central letter. Since there are four possible central letters and four possible distractor letters, there are 16 possible combinations of central and distractor letters, and each combination appeared an equal number of times in each block (nine repetitions yielding 144 flanker trials per block).

On two of the four blocks, the distractors were located toward the upper left and bottom right of the central letter, and on the other two blocks the distractors were located toward the upper right and lower left. The center of the distractors was 1.58 (1.122degrees of vertical visual angle) degrees of visual angle from the center of the central letter. Critically, within a block, the flanker configurations never changed. Letters were displayed in 14-point Arial font. The central letter was white [255, 255, 255]. As mentioned earlier, there were two versions of this task. In each version the contrast of the distractor letters relative to the background were different. In the equal-contrast version, the distractor letters were white [255, 255, 255], the same color as the central target. In the low-contrast version, the distractor letters were a light gray that was defined as 25% of the difference (rounded up) between the background color and white [139, 139, 139]. The phenomenological effect is that in the low-contrast version of the task, the distractors are harder to see because they blend in more with the background.

The two-dot displays consisted of two white dots (one pixel wide) that, during the main experiment, appeared simultaneously at the two possible top distractor positions (1.58 degrees of visual angle from the center of the screen to the top left or the top right). Thus, one of the dots appeared in the same location as the “upper” distractor letters in the flanker task in that block. Participants were instructed to judge which dot they perceived to appear first. To indicate that the left dot appeared first, they would press the *S* key. To indicate that the right dot appeared first, they would press the *K* key. To enhance the likelihood participants will make a genuine attempt to judge the temporal order, during the practice trials one of the two dots would appear 50 ms prior to the second dot. However, during the actual experimental run, the two dots appeared simultaneously.

Every trial began with 500 ms of a fixation cross presented at the center of the screen. The fixation cross was black [0, 0, 0]. Next, there was a 500-ms blank interval. Finally, the appropriate stimulus (flanker display or dots, depending on the trial) was displayed until a response was made. Participants were given the chance to rest in between blocks for as long as they wanted, though no one took a break for more than a few minutes (<5 min). Each session began with 20 practice trials consisting of 16 flanker trials and four dot trials. During the practice, participants received visual feedback such that if they made an error on the flanker task, after their response the following fixation cross would turn red for the first 250 ms it was displayed, and then turn black for another 250 ms.

## Experiment 1: Results

### Flanker-task performance

Response time in ms (RT) and accuracy rate (i.e., proportion of correct responses) were measured as dependent variables for the flanker task. The response-time data was cleaned to account for outliers (±2 *SD*s). Cutoff points were calculated independently for congruent and incongruent responses for each participant. Within these conditions, the outlier analysis was conducted at the smallest cell level. For the younger participants, this resulted in the loss of an average of 4.41% (*SEM* = .24%) of the equal-contrast response time data and 4.39% (.22%) of the low-contrast response-time data, per participant. For the older participants, this resulted in the loss of an average of 4.30% (*SEM* = .22%) of the equal-contrast response-time data and 4.30% (.19%) of the low-contrast response-time data, per participant. All results reported as mean ± standard error of the mean (*SEM*).

**Fig. 2:**
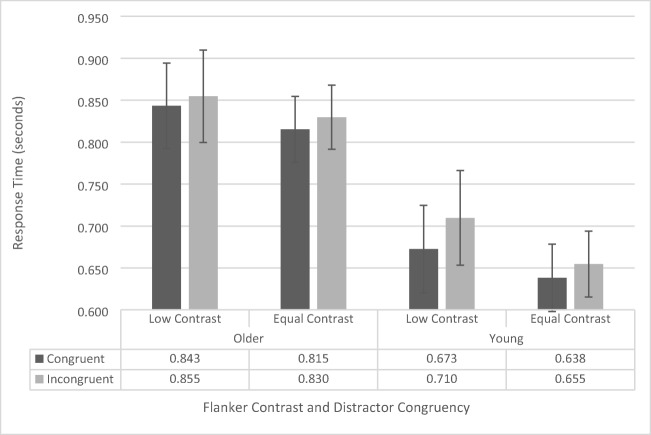
Flanker-task response-time data as a function of congruency, flanker configuration, and age group. Error bars reflect standard error of the mean

Response-time data were analyzed using a repeated-measures ANOVA, with contrast (low vs. regular), flanker configuration (upper left/lower right vs. upper right/lower left), and congruency (congruent vs. incongruent) as within-subjects factors, and age group (younger vs. older) as a between-subjects factor. As there was no main effect of flanker configuration, *F*(1, 41) = .711, *p* = .404, and no significant interactions with flanker configuration (all *p*s > .261, all *F*s < 1.298), we collapsed the data across flanker configuration and conducted a repeated-measures ANOVA including contrast, congruency, and age group (see Fig. 2).

The analysis revealed a significant main effect of congruency, *F*(1, 41) = 18.697, *p* < .001, ${\eta}_p^2$= .313, as across both groups participants responded faster for congruent trials (742 ms ± 31 ms) than for incongruent trials (762 ms ± 32 ms). There was also a significant main effect of age group, *F*(1, 41) = 7.004, *p* = .011, ${\upeta}_{\mathrm{p}}^2$= .146, as younger participants were generally faster (669 ms ± 44 ms) than the older participants (836 ms ± 45 ms) were. The main effects of contrast, *F*(1, 41) = 3.289, *p* = .077, approached, but was not significant (low contrast: 770 ms ± 38 ms, equal contrast: 734 ms ±28 ms). None of the interactions were significant (all *p*s > 0.136, all *F*s < 2.310).

**Fig. 3:**
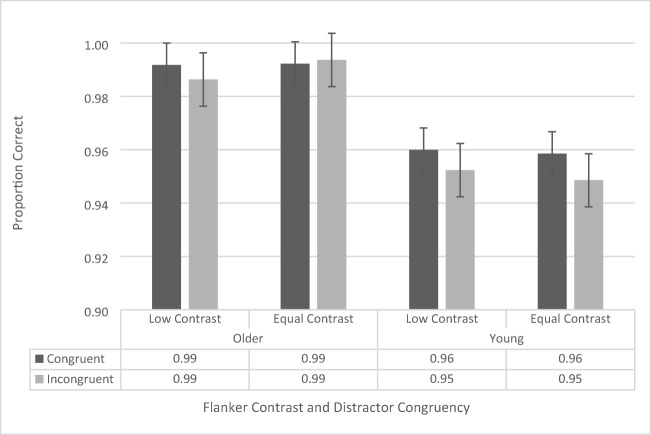
Flanker-task accuracy data as a function of congruency, flanker configuration, distractor contrast, and age group. Error bars reflect standard error of the mean

The accuracy data were analyzed using a repeated-measures ANOVA, with contrast (low vs. regular), flanker configuration (upper left/lower right vs. upper right/lower left), and congruency (congruent vs. incongruent) as within-subjects factors, and age group (younger vs. older) as a between-subjects factor. There was no main effect of flanker configuration, *F*(1, 41) = 2.734, *p* = .106, and there were no significant interactions with flanker configuration, although it should be noted that the four-way interaction approached significance, *F*(1, 41) = 3.164, *p* = .083 (all other *p*s > .196 and all other *F*s < 1.728). Therefore, we collapsed the data across flanker configuration and conducted a repeated-measures ANOVA including contrast, congruency, and age group (see Fig. 3).

The repeated-measures ANOVA revealed a significant main effect of congruency, *F*(1, 41) = 8.61*, p* = .005,${\upeta}_{\mathrm{p}}^2$= .174, as participants across both groups were more accurate during congruent trials (97.6% ± 0.3%) than during incongruent trials (97.0% ± 0.4%). The analysis also revealed a significant main effect of age group, *F*(1, 41) = 28.786, *p* < .001, ${\upeta}_{\mathrm{p}}^2$= .412, with the older participants performing more accurately (99.1% ± 0.5%) than the younger ones (95.5% ± 0.5%). The main effect of contrast, *F*(1, 41) = .036, *p* = .851, was not significant. The two-way interaction between age group and congruency approached significance, *F*(1, 41) = 3.343, *p* = .075, as the congruency effect for the younger participants (congruent: 95.9% ± 0.5%, incongruent: 95.0% ± 0.5%) trended toward being larger than that of the older participants (congruent: 99.2% ± 0.4%, incongruent: 99.0% ± 0.5%). None of the other interactions were significant (all *p*s > .128, all *F*s < 2.412).

### Temporal-order-judgment task

**Fig. 4:**
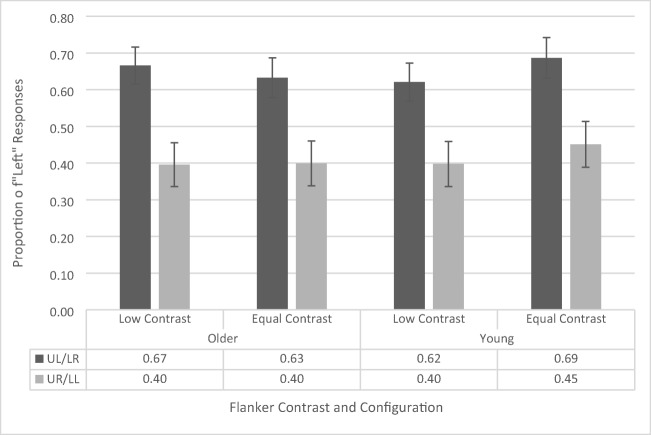
Proportion of “left” responses on temporal-order-judgement task as a function of flanker configuration, distractor contrast, and age group. UL/LR refers to upper left/lower right and UR/LL refers to upper right/lower left. Error bars reflect standard error of the mean

The AWB effect (see Fig. 4) is indexed by the difference in likelihood of identifying the left or right dots as appearing first as a function of flanker position. Thus, we have used a repeated-measures ANOVA (similar to Tsal & Makovski, 2006) on the proportion of “left” responses with contrast (low vs. equal) and flanker configuration (upper left vs. upper right) as within-subjects factors and age group (younger vs. older) as a between-subjects factor (see Fig. 4). The ANOVA revealed a main effect of flanker configuration, *F*(1, 41) = 26.056, *p* < .001, ${\eta}_p^2$= .389, as participants across both groups had a greater proportion of left responses when a flanker appeared in the upper left position (65.2% ± 3.4%) compared with the upper right position (41.1% ± 4.2%). This is the standard AWB effect. The main effects of contrast, *F*(1, 41) = 1.48, *p* = .231, and age group, *F*(1, 41) = .067, *p* = .796, were not significant. There is a common misconception that simple main effects cannot be interpreted if there is a nonsignificant interaction. However, this is not the case. According to Wei, Carroll, Harden, and Wu (2012), the simple effects are a reasonable and interpretable analysis when there is a nonsignificant interaction given that the main effect of one of the variables is significant. As the main effect of flanker configuration is significant, and the Age × Flanker Configuration interaction is the primary effect of interest in this study, we further confirmed these results by calculating simple main effects, which revealed that both the younger (upper left flanker: 65.4% ± 4.9%, upper right flanker: 42.4% ± 6.0%, *p* = .001) and older (upper left flanker: 64.9% ± 4.8%, upper right flanker: 39.8% ± 5.8%, *p* < .001) cohorts independently showed a significant white-bear effect.

A marginally significant interaction between contrast and age group was also found, *F*(1, 41) = 4.079, *p* = .05, ${\eta}_p^2$= .090. Analysis of simple main effects revealed that this was driven by a lower proportion of left responses in the low-contrast condition (50.9%, 4.6%) than in the equal-contrast condition (56.9%, 4.3%) for the younger participants (*p* = .029), but no difference in proportion of left responses between low (53.1%, 4.5%) and equal (51.6%, 4.2%) contrast conditions for the older participants (*p* = .569). This interaction suggests that younger participants were more sensitive to the contrast manipulation. However, since this was not with respect to the flanker configuration, it does not reflect AWB-related processes. Indeed, two-tailed one-sample *t* tests revealed that, for the younger participants, the overall proportion of left responses (across both flanker configurations) in the low-contrast condition, *t*(19) = .177, *p* = .861, and the equal-contrast condition, *t*(19) = 1.63, *p* = .119, did not differ significantly from .50 (i.e., chance performance). This supports the notion that the increased tendency of the younger participants to respond left during the equal-contrast trials cannot account for the white-bear effect in that condition. The remaining interactions did not reach significance levels (all *p*s > .551, all *F*s < .361).

This data suggest that both younger and older participants exhibit the standard white-bear effect and that the magnitude of the effect is consistent across age groups. To further interrogate this result, we conducted a Bayesian repeated-measures ANOVA (using JASP; Jarosz & Wiley, 2014; Marsman & Wagenmakers, 2017; Wagenmakers, 2007) and calculated the inclusion Bayes factor for each main effect and interaction. The inclusion Bayes factor is a measure of the extent to which a factor of interest should be included in a model of the data, compared with all other models. The inclusion Bayes factor for the main effect of flanker configuration was 1.197e+9, indicating that there is very strong/decisive evidence in favor of a model that includes flanker configuration to explain the data. However, the inclusion Bayes factors provides less than weak/anecdotal evidence for all other main effects and interactions (all BF_Inclusion_ < .138; it should be noted that the terms very strong/decisive and weak/anecdotal are based on guidelines detailed in Jarosz & Wiley, 2014). This supports the conclusion that in Experiment 1, both younger and older participants exhibited a white-bear effect with a similar magnitude.

## Experiment 1: Discussion

We found no age difference in the temporal-order-judgment task (the AWB statistic). Across both younger and older participants, a similar tendency was observed to perceive the dot appearing in an expected flanker position as appearing first. At first glance, proactive allocation of attention prior to inhibition appears to be intact in aging populations. As expected, interference from the flankers was observed in both RTs and accuracy (main effect of congruency) for all participants regardless of age. The group difference in RT was also expected, with older participants typically responding overall slower. The group difference in accuracy with older participants performing overall more accurately than younger participants is also quite common in the aging literature. These findings may represent a simple speed–accuracy trade-off in the two groups or, alternatively, may be attributable to a generalized age-related deficit in processing speed (Salthouse, 2000; Salthouse & Meintz, 1995; Verhaeghen & De Meersman, 1998). Critically, the data did not point to increased interference in old age in this task and, if anything, the marginally significant interaction between age and congruency reported for the accuracy data suggested more interference in younger compared with older participants.

Although the lack of a between-group inhibition deficit is arguably unexpected given the ample evidence that proactive inhibition is impaired in aging (Jimura & Braver, 2010; Paxton et al., 2008), it does fit with previous studies using the flanker task in older participants who did not show increased interference in older participants (Fernandez-Duque & Black, 2006; Kawai, Kubo-Kawai, Kubo, Terazawa, & Masataka, 2012; Salthouse, 2010; Verhaeghen, 2011). One possibility is that flanker tasks are not sensitive enough to detect age-related differences in inhibition, as a number of previous studies using this task have also failed to find group differences. For instance, Salthouse (2010) found no age-related behavioral differences in flanker-task performance and questioned its utility as a measure of executive function and inhibition (also see Fernandez-Duque & Black, 2006; Kawai et al., 2012; Verhaeghen, 2011). A further possibility for the comparable congruency effects here could be that performance in the flanker task reflects reactive (rather than proactive) inhibition, which is supposedly intact in old age (Braver, Satpute, Rush, Racine, & Barch, 2005; Jimura & Braver, 2010; Paxton et al., 2008).

That being said, a review of our study and others revealed that the studies that fail to find age differences may have been not sensitive to differences in inhibition because the task was simply too easy. In these tasks the flanker stimuli were presented for up to 2,000 ms (Kawai et al., 2012), 1,500 ms (Salthouse, 2010, Study 1), or 500 ms (Fernandez-Duque & Black, 2006; in this study, the flanker was also combined with a cueing task). In our study, the flanker stimuli were presented until a response was made. Although this was consistent with the original AWB studies, this may not be the appropriate timing for this study where we are interested in proactive allocation of attention prior to inhibition. Under these long presentation times, even though proactive inhibition is clearly taking place (i.e., the white-bear effect), subsequent inhibition of the flankers may not be necessary, because there is enough time to process the flankers and the target sequentially, rather than simultaneously. Therefore, in the next set of experiments we assessed the possibility that long presentation times may be making flanker tasks and the AWB task not sensitive to age-related inhibition deficits. We conducted the same attentional-white-bear study across younger and older participants, but this time the presentation time for the flanker stimuli was limited to either 100 ms or 200 ms.

## Experiments 2a and 2b: Methods

### Participants

Twenty-one younger and 21 older participants took part in this study, each completing four successive behavioral experiments (low and regular contrast with 200 ms or 100 ms presentation); however, because of poor performance (>20% invalid button presses during the temporal-judgement task in either presentation time condition), one older participant was excluded from the study entirely. One younger participant was excluded from the 200-ms analysis. One additional older participant and a different younger participant was excluded from the 100-ms analysis. For the 200-ms experiments, 20 younger participants (*M*_age_ = 25.10 years, *SEM*_age_ = 0.746, age range: 20–32 years, 17 females) and 20 older participants (*M*_age_ = 71.75, *SEM*_age_ = .99, age range: 65–81 years, 13 females) were included in the analysis. For the 100-ms experiments, 20 younger participants (*M*_age_ = 24.85 years, *SEM*_age_ = 0.805, age range: 19–32 years, 17 females) and 19 older participants (*M*_age_= 71.57 years, *SEM*_age_ = 1.03, age range: 65–81 years, 12 females) were included in the analysis. Younger participants were recruited from the undergraduate population in the School of Psychology at the University of Birmingham, UK. They were compensated for their participation with course credits. The older participants were recruited from a volunteer pool maintained by the School of Psychology at the University of Birmingham. They were compensated for 1.5 hours of their time with a one-time payment of £7. All participants had to sign an informed consent form prior to the study. Participants’ were healthy with no history of head injury, mental health issues, or neurological disorders. The older participants were screened for decline in cognitive functions using the Montreal Cognitive Assessment (MoCA). All of the older participants scored within the normal range (>26).

### Stimuli and procedure

The stimuli and procedure for this study were nearly identical to the procedure described in the Methods section of Experiment 1. The only differences were that flanker stimuli were presented for either 100 ms or 200 ms, instead of until response. After the presentation time was up, the screen displayed a fixation cross until a response was made. The stimulus timing condition was blocked, and conditions were presented to participants in the following order: low-contrast flankers with 200-ms presentation time; equal-contrast flankers with 200-ms presentation time; low-contrast flankers with 100-ms presentation time; equal-contrast flankers with 100-ms presentation time. This order was selected so that participants could not establish strategies during the faster 100-ms condition that they could then easily deploy during the 200-ms condition.

## Experiment 2a: 200-ms presentation-time results

### Flanker-task performance

Response time (RT) in ms and accuracy rate (i.e., proportion of correct responses) were measured as dependent variables for the flanker task. The response-time data were cleaned to account for outliers (±2 *SD*s). Cutoff points were calculated independently for congruent and incongruent responses for each participant. Within these conditions, the outlier analysis was conducted at the smallest cell level. For the younger participants, this resulted in the loss of an average of 4.38% (*SEM* = .21%) of the equal-contrast response-time data and 3.94% (.39%) of the low-contrast response-time data, per participant. For the older participants, this resulted in the loss of an average of 3.73% (*SEM* = .35%) of the equal-contrast response-time data and 3.78% (.25%) of the low-contrast response-time data, per participant. All results reported as mean ± standard error of the mean (*SEM*).

**Fig. 5:**
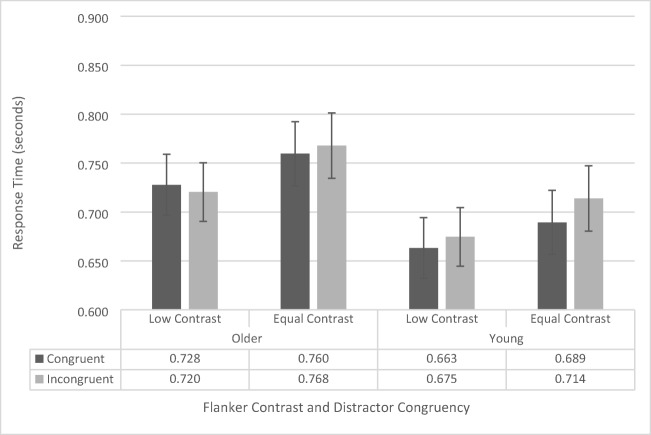
200-ms stimulus presentation-time flanker-task response-time data as a function of congruency, flanker contrast, and age group. Error bars reflect standard error of the mean

Response-time data were analyzed using a repeated-measures ANOVA, with contrast (low vs. regular), flanker configuration (upper left/lower right vs. upper right/lower left), and congruency (congruent vs. incongruent) as within-subjects factors, and age group (younger vs. older) as a between-subjects factor. As there was no main effect of flanker configuration, *F*(1, 38) = .840, *p* = .365) and no significant interactions with flanker configuration (all *p*s > .145, all *F*s < 2.214), we collapsed the data across flanker configuration and conducted a repeated-measures ANOVA including contrast, congruency, and age group (see Fig. 5).

The main effect of congruence was significant, *F*(1, 38) = 4.27, *p* = .045, ${\eta}_p^2$= .101, with participants responding faster on congruent (710 ms ± 22 ms) trials than to incongruent (719 ms ± 21 ms) trials. The main effect of age group was not significant, *F*(1, 38) = 1.81, *p* = .186. The interaction between congruence and age group approached significance, *F*(1, 38) = 3.87, *p* = .057, ${\eta}_p^2$= .092. To further investigate this potential effect, we assessed simple main effects, which revealed that this effect is driven by a significant congruency effect for younger participants (*p* = .007; congruent RT: 676 ms ± 31 ms; incongruent RT: 694ms ± 31 ms), but no significant difference in congruence for the older participants, who showed virtually identical RTs for congruent (744 ms ± 31 ms) and incongruent (744 ± 31 ms) displays. The interaction between contrast and congruence also approached significance, albeit to a lesser degree, *F*(1, 38) = 3.006, *p* = .091,${\eta}_p^2$= .073. Simple main effects revealed this was driven by a significant congruency effect during regular-contrast trials (*p* = .007; congruent RT: 725 ms ± 23 ms; incongruent RT: 741 ms ± 24 ms), but no significant difference in congruence during low-contrast trials (*p* = .750; congruent RT: 696 ms ± 22 ms; incongruent RT: 698 ms ± 21 ms). The interaction between contrast and age group, *F*(1, 38) = .130, *p* = .720, and the three-way interaction, *F*(1, 38) = .024, *p* = .878, were not significant.

**Fig. 6:**
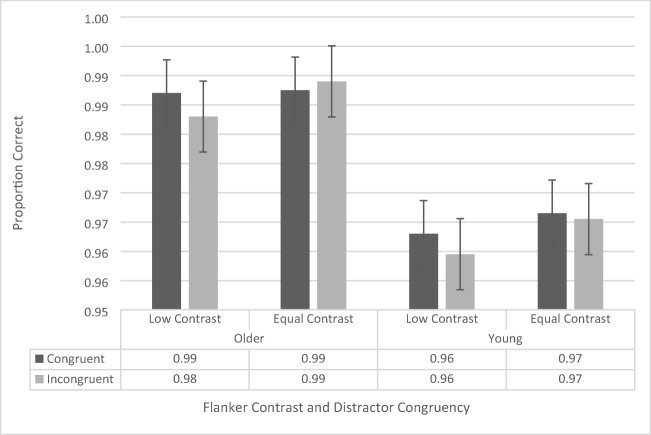
200-ms stimulus presentation-time flanker-task proportion-correct data as a function of congruency, flanker contrast, and age group. Error bars reflect standard error of the mean

For the accuracy data, we conducted a repeated-measures ANOVA, with contrast (low vs. regular), flanker configuration (upper left/lower right vs. upper right/lower left), and congruency (congruent vs. incongruent) as within-subjects factors and age group (younger vs. older) as a between-subjects factor. As there was no main effect of flanker configuration, *F*(1, 38) = .899, *p* = .349, or significant interactions with flanker configuration (all *p*s > .284, all *F*s < 1.183), we collapsed the data across flanker configuration and conducted a repeated-measures ANOVA including contrast, congruency, and age group (see Fig. 6).

The ANOVA revealed a significant main effect of age group, *F*(1, 38) = 7.954*, p* = .008,${\eta}_p^2$= .173, in which older participants (98.7% ± 0.6%) were more accurate than younger participants (96.4% ± 0.6%) were. The main effect of contrast, *F*(1, 38) = 1.90, *p* = .176, and congruence, *F*(1, 38) = .844*, p* = .364, were not significant. None of the interactions were significant (all *p*s > .323, all *F*s < 1.002).

### Temporal-order-judgment task

**Fig. 7:**
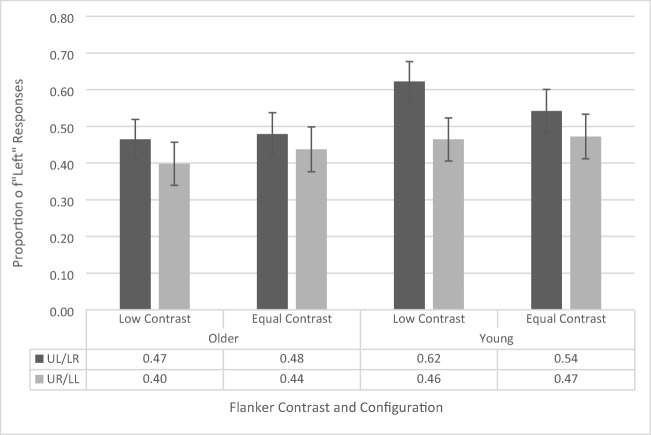
Proportion of “left” responses on temporal-order-judgement task with 200-ms flanker-presentation time as a function of flanker configuration, flanker contrast, and age group. UL/LR refers to upper left/lower right and UR/LL refers to upper right/lower left. Error bars reflect standard error of the mean

Next, we assessed the presence and magnitude of the attentional-white-bear effect (see Fig. 7). We conducted a repeated-measures ANOVA on the proportion of “left” responses, with contrast (low vs. equal) and flanker configuration (upper left vs. upper right) as within-subjects factors, and age group (younger vs. older) as a between-subjects factor. The ANOVA revealed a main effect flanker configuration, *F*(1, 38) = 7.196, *p* = .011, ${\eta}_p^2$= .159, as participants across both groups had a greater proportion of left responses when a flanker appeared in the upper left position (52.7% ± 3.6%) compared with the upper right position (44.3% ± 3.9%). This is the standard AWB effect. The main effects of contrast, *F*(1, 38) = .041, *p* = .840, and age group, *F*(1, 38) = 1.358, *p* = .251, were not significant. In contrast to Experiment 1, there were no significant interactions (all *p*s > .185, all *F*s < 1.823). To be consistent with our analysis of Experiment 1 and also to further investigate our effect of interest, we calculated the simple main effects of the Age × Distractor Location interaction (Note: The Age × Flanker Configuration interaction is nonsignificant, but the main effect of flanker configuration is significant). This analysis revealed that the younger participants (upper left flanker: 58.2% ± 5.2%; upper right flanker: 46.8% ± 5.6%; *p* = .014) showed a significant white-bear effect, but the older participants did not (upper left flanker: 47.2% ± 5.2%; upper right flanker: 41.8% ± 5.6%; *p* = .228).

However, given the disagreement between the nonsignificant interaction and the simple main effects, this result must be interpreted with care. To further assess this potential effect, we calculated the Bayes factor in support of the alternative hypothesis that there is an interaction between age group and configuration. First, we used JASP to compare the Bayes factor for a model that included just the main effects of age group and flanker configuration (BF_10_ = 12.669) and for a model that included the main effects and their interaction (BF_10_ = 5.138). Next, we divided the model with the interaction by the model without the interaction (5.138/12.669 = 0.405). This suggests that the observed data are 0.405 times more likely to occur under a model where there is an interaction between age group and configuration (compared with a model where there is no interaction). Consistent with the null hypothesis significance testing, the Bayes factor analysis essentially provides no evidence in favor of the interaction.

## Experiment 2b: 100-ms presentation-time results

### Flanker-task performance

Response time (RT) in ms and accuracy rate (i.e., proportion of correct responses) were measured as dependent variables for the flanker task. The response-time data were cleaned to account for outliers (±2 *SD*s). Cutoff points were calculated independently for congruent and incongruent responses for each participant. Within these conditions, the outlier analysis was conducted at the smallest cell level. For the younger participants, this resulted in the loss of an average of 4.23% (*SEM* = .22%) of the equal-contrast response-time data and 4.18% (.30%) of the low-contrast response-time data, per participant. For the older participants, this resulted in the loss of an average of 3.95% (*SEM* = .29%) of the equal-contrast response-time data and 3.455% (.26%) of the low-contrast response-time data, per participant. All results reported as mean ± standard error of the mean (*SEM*).

**Fig. 8:**
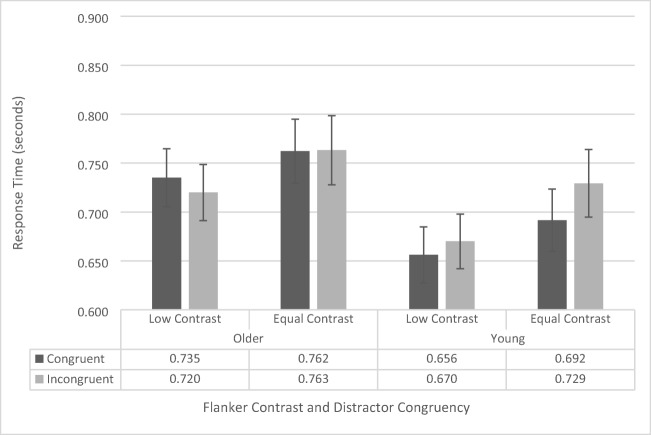
100-ms stimulus presentation-time flanker-task response-time data as a function of congruency, flanker contrast, and age group. Error bars reflect standard error of the mean

Response-time data were analyzed using a repeated-measures ANOVA, with contrast (low vs. regular), flanker configuration (upper left/lower right vs. upper right/lower left), and congruency (congruent vs. incongruent) as within-subjects factors, and age group (younger vs. older) as a between-subjects factor. Although there was a main effect of flanker configuration, *F*(1, 38) = 5.70, *p* = .022, ${\eta}_p^2$= .134, driven by faster responses when the flanker appeared in the upper left/lower right (725 ms ± 22 ms) compared with the upper right/lower left (707 ms ± 21 ms), there were no significant interactions with flanker configuration (all *p*s > .265, all *F*s < 1.283). Therefore, we collapsed the data across flanker configuration and conducted a repeated-measures ANOVA including contrast, congruency, and age group (see Fig. 8).

The analysis revealed that there was a main effect of contrast, *F*(1, 38) = 11.407, *p* = .002, ${\eta}_p^2$= .236, as across both groups’ participants responded faster when presented with low-contrast flankers (695 ms ± 20 ms) than with equal-contrast flankers (737 ms ± 24 ms). The main effect of congruence was also significant, *F*(1, 38) = 5.754, *p* = .022, ${\eta}_p^2$= .135, as participants responded faster on congruent trials (711 ms ± 21 ms) than on incongruent trials (721 ms ± 21 ms). However, these were qualified by an interaction between contrast and congruence, *F*(1, 38) = 5.582, *p* = .024, ${\eta}_p^2$= .131. Simple main effects revealed that this was driven by faster responses on congruent trials when presented with equal-contrast flankers (*p* = .002; congruent: 727 ms ± 23 ms; incongruent: 746 ms ± 25 ms), but no difference in congruency when presented with low-contrast flankers (*p* = .907; congruent: 696 ms ± 21 ms; incongruent: 695 ms ± 20 ms). The main effect of age group was not significant, *F*(1, 38) = 1.916, *p* = .175, but this too was qualified by an interaction between age group and congruence, *F*(1, 38) = 17.769, *p* < .001, ${\eta}_p^2$= .324. Simple main effects revealed that, like in the 200-ms experiments, this interaction was driven by a significant congruency effect for younger participants (*p* < .001; congruent RT: 674 ms ± 29 ms; incongruent RT: 700 ms ± 30 ms), but no significant difference in congruence for the older participants (*p* = .213; congruent RT: 749 ms ± 30 ms; incongruent RT: 742 ms ± 30 ms). Neither the interaction between contrast and group nor the three-way interaction were significant (both *p*s > .619).

Accuracy data were analyzed using a repeated-measures ANOVA, with contrast (low vs. regular), flanker configuration (upper left/lower right vs. upper right/lower left), and congruency (congruent vs. incongruent) as within-subjects factors, and age group (younger vs. older) as a between-subjects factor. The main effect of flanker configuration was not significant, *F*(1, 38) = .114, *p* = .738, but there was a marginally significant four-way interaction, *F*(1, 38) = 4.148, *p* = .049,${\eta}_p^2$= .101. To assess if this interaction is meaningful, we compared the Bayes factor of a model with and without the four-way interaction (BF_10_-with/BF_10_-without = .011/.006 = 1.833). The Bayes factor (BF_10_) is a measure of how likely a set of data is under the alternative hypothesis compared with the null hypothesis. This suggested that the data were 1.833 times more likely under a model with the four-way interaction than without, constituting weak or anecdotal evidence (Jarosz & Wiley, 2014) in favor of the four-way interaction.

**Fig. 9:**
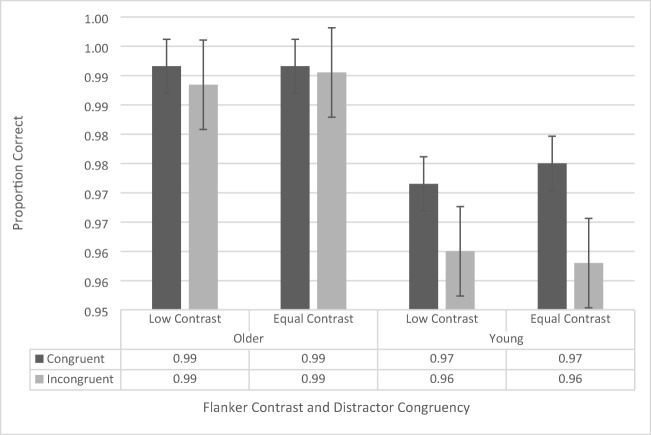
100-ms stimulus presentation-time flanker-task proportion-correct data as a function of congruency, flanker contrast, and age group. Error bars reflect standard error of the mean

Given the limited evidence that the four-way interaction is meaningful, and to be consistent with the other analyses throughout the paper, we collapsed the data across flanker configuration and conducted a repeated-measures ANOVA including contrast, congruency, and age group (see Fig. 9). The ANOVA revealed a significant main effect of age group, *F*(1, 38) = 12.815, *p* = .001,${\eta}_p^2$= .257, in which older participants (99.1% ± 0.5%) were more accurate than in younger participants (96.6% ± 0.5%). There was also a main effect of congruence, *F*(1, 38) = 13.817, *p* = .001,${\eta}_p^2$= .272) as participants were more accurate on congruent trials (98.2% ± 0.3%) than on incongruent trials (97.4% ± 0.4%). However, these effects were qualified by a significant interaction between age group and congruence, *F*(1, 38) = 7.619, *p* = .009,${\eta}_p^2$= .171. Simple main effects revealed that this was driven by better performance in the congruent condition (97.3% ± 0.4%) than in the incongruent condition (95.9% ± 0.6%) for the younger participants (*p* < .001), but no difference in performance (congruent: 99.2% ± 0.4%; incongruent: 98.9% ± 0.6%) for the older participants (*p* = .508). The main effect of contrast, *F*(1, 38) = .165, *p* = .687, and none of the interactions were significant (all *p*s > .252).

### Temporal-order-judgment task

**Fig. 10:**
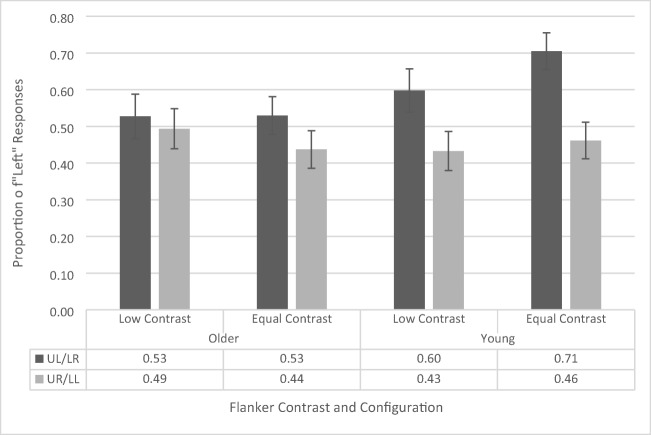
Proportion of “left” responses on the temporal-order-judgement task with 100-ms flanker presentation time as a function of flanker configuration, flanker contrast, and age group. UL/LR refers to upper left/lower right and UR/LL refers to upper right/lower left. Error bars reflect standard error of the mean

A repeated-measures ANOVA (see Fig. 10) on the proportion of “left” responses, with contrast (low vs. equal) and flanker configuration (upper left vs. upper right) as within-subjects factors and age group (younger vs. older) as a between-subjects factor (see Fig. 9). The ANOVA revealed a main effect of flanker configuration, *F*(1, 38) = 12.896, *p* = .001, ${\eta}_p^2$= .258, as participants across both groups had a greater proportion of left responses when a flanker appeared in the upper left position (59.0% ± 3.7%) compared with the upper right position (45.6% ± 3.5%). This is the standard AWB effect.

The main effects of contrast, *F*(1, 38) = 1.344, *p* = .254, and age group, *F*(1, 38) = .727, *p* = .399, were not significant. However, this was qualified by a significant interaction between contrast and age group, *F*(1, 38) = 7.317, *p* = .010,${\eta}_p^2$= .165. Simple main effects revealed that this interaction was driven by a lower proportion of left responses in the low-contrast condition (51.6% ± 4.8%) than in the equal-contrast condition (58.3% ± 4.1%) for the younger participants (*p* = .009), but no difference in proportion of “left” responses between low (51.1% ± 4.9%) and equal (48.3%± 4.2%) contrast conditions for the older participants (*p* = .287). This is the same effect that was found in Experiment 1, but not in Experiment 2a, suggesting that younger participants may be more sensitive to the contrast manipulation. A two-tailed one-sample *t* test revealed that, for the younger subjects, the overall proportion of left responses (across both flanker configurations) in the low-contrast condition, *t*(19) = −.340, *p* = .738, did not differ significantly from .50 (i.e., chance performance). However, the overall proportion of left responses did differ in the equal-contrast condition, *t*(19) = 2.35, *p* = .029, *d* = .52, suggesting that younger participants exhibited a bias for responding “left” during the equal-contrast trials. However, since this effect is not with respect to the flanker configuration, it does not reflect AWB-related processes and does not affect our ability to interpret AWB effects.

Critically, the interaction between flanker configuration and age group approached significance, *F*(1, 38) = 3.611, *p* = .065,${\eta}_p^2$= .089. We further assessed this interaction with simple main effects since the main effect of flanker configuration was significant (Wei et al., 2012). Simple main effects revealed that younger participants appeared to respond left more often when flankers were in the upper left (65.1% ± 5.2%) compared with the upper right (i.e., the standard white-bear effect; *p* < .001; 44.7% ± 4.9%), whereas older participants showed no difference in left responses due to flanker configuration (*p* = .245; 52.8% ± 5.3%; 46.6% ± 5.0%, for upper left and upper right flankers, respectively). However, given the nonsignificant interaction, this result must be interpreted with care. To further assess this potential effect, we calculated the Bayes factor in support of the alternative hypothesis that there is an interaction between age group and configuration. First, we used JASP to compare the Bayes factor for a model that included just the main effects of age group and flanker configuration (BF_10_ = 3,308.896) and for a model that included just the main effects and their interaction (BF_10_ = 17,518.914). Next, we divided the model with the interaction by the model without the interaction (17,518.914/3,308.896 = 2.94). This suggests that the observed data is 2.94 times more likely to occur under a model where there is an interaction between age group and configuration (compared with a null model). According to Jarosz and Wiley (2014), this value is right on the cutoff between being considered weak/anecdotal evidence (<3) or positive/substantial evidence (>3).

The interaction between contrast and flanker configuration, *F*(1, 38) = 3.266, *p* = .079, as well as the three-way interaction, *F*(1, 38) = .070, *p* = .792, were not significant.

### Allocation versus inhibition

In the 100-ms condition, older participants showed differences in both allocation of attention to expected distractors (absence of a white-bear effect) and inhibition of these distractors (absence of a congruency effect). Here, we tested if there was a relationship between these two variables. To reflect the allocation of attention, we calculated a white-bear metric, which was the difference in “left” responses between flankers in the upper left or upper right corner of the stimuli. Higher values of this metric reflect a larger white-bear effect and presumably more allocation of attention toward the expected flankers prior to their occurrence. To reflect the inhibition of attention, we calculated the response-time congruency effect, which was the difference in response time between congruent and incongruent trials (Incongruent − Congruent). Higher values of this metric reflect a larger congruency effect and ostensibly less inhibition of the distractors. It should be noted here that the white-bear and congruency effects reflect performance on different trials with distinct tasks (temporal order judgment vs. flanker), therefore this is an indirect measure of the relationship between attention allocation and inhibition. We calculated these values separately for the low-contrast and equal-contrast conditions.

**Fig. 11:**
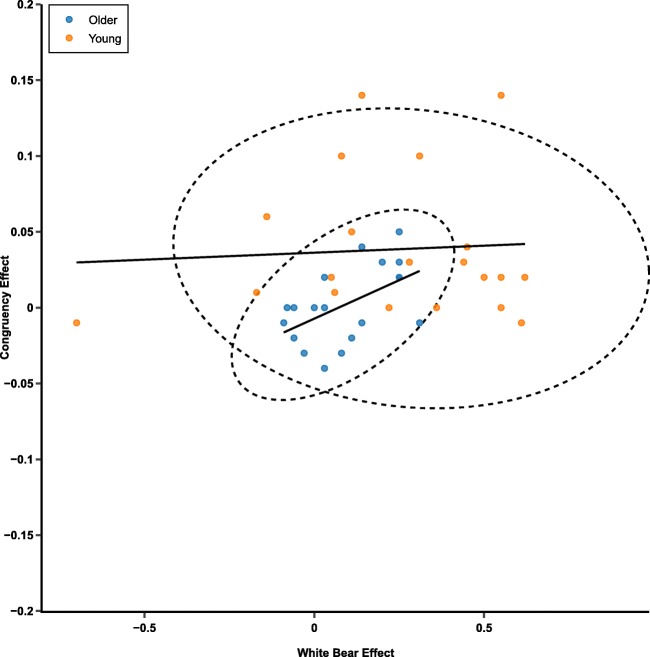
Scatterplot reflecting the white bear effect and the congruency effect for equal-contrast flankers presented for 100 ms. Larger values of the congruency effect reflect worse inhibition. Larger values of the white bear statistic reflect more allocation of attention. The dotted lines reflect 95% confidence ellipses based on a *t* distribution. (Color figure online)

For the low-contrast trials, the correlation between the white-bear metric and the congruency effect was not significant for both the younger, *r*(18) = .011, 95% CI_Upper_ = .452, 95% CI_Lower_= −.433, *p* = .962, and older, *r*(17) = −.342, 95% CI_Upper_ = .133, 95% CI_Lower_ = −.689, *p* = .151, participants. For the equal-contrast trials, the correlation was not significant for the younger participants, *r*(18) = .065, 95% CI_Upper_= .494, 95% CI_Lower_ = −.388, *p* = .784, but it was significant for the older participants, *r*(17) = .524, 95% CI_Upper_ = .790, 95% CI_Lower_ = .092, *p* = .021 (see Fig.11). This correlation suggests that for the older participants, a larger white-bear effect was associated with a larger congruency effect. In other words, more effective proactive allocation of attention in the temporal-order-judgment task was associated with less effective inhibition in the flanker task.

However, these results must be interpreted with care because a sample size of 21 per group is small for a correlational analysis. To further assess this effect, we used JASP to calculate the Bayes factor (Jarosz & Wiley, 2014; Marsman & Wagenmakers, 2017; Wagenmakers, 2007) for the equal-contrast correlation results for the younger and older cohort independently. The Bayes factor is a measure of how much more likely is a set of data under the alternative or null hypothesis. For the older participant data, the alternative hypothesis is a positive correlation, and the null hypothesis is no correlation. The Bayes factor in favor of the alternative hypothesis was 6.625, suggesting that the observed data is 6.625 times more likely to occur under a model where there is a positive correlation between the congruency effect and the white-bear effect than one in which there is no correlation. This is considered positive or substantial evidence in favor of the alternative hypothesis (a positive correlation; based on guidelines detailed in Jarosz & Wiley, 2014). For the younger participant data, we calculated the inverse Bayes factor such that the alternative hypothesis is no correlation and the null hypothesis is any (positive or negative) correlation. The inverse Bayes factor in favor of the alternative hypothesis was 3.488, suggesting that the observed data is 3.488 times more likely to occur under a model where there is no correlation between the congruency effect and the white-bear effect than one in which there is a correlation (positive or negative). This value is right on the cutoff between being considered weak/anecdotal evidence (<3) or positive/substantial evidence (>3; based on guidelines detailed in Jarosz & Wiley, 2014).

Although the Bayes factor analysis does increase our confidence in the correlation for the older participants, because of the sample size, the correlation analysis is exploratory/speculative at best. It does, however, suggest that future studies of the attentional-white-bear paradigm should assess the relationship between allocation and inhibition of attention with a larger sample size to confirm or disconfirm these findings and better understand the assumed relationship between allocation and inhibition in the attentional-white-bear paradigm (and in some inhibition processes in general).

## Experiment 2a and 2b: Discussion

When the flanker stimuli were presented for 200 ms, both younger and older participants exhibited a white-bear effect of similar magnitude, similar to Experiment 1. However, in contrast to Experiment 1, only the younger participants exhibited a congruency effect (faster RTs on congruent trials). Furthermore, older participants were more accurate than younger participants. While increased accuracy in aging populations is often attributed to speed–accuracy trade-offs, in this case there was no difference in overall response time between the age groups, making this interpretation unlikely. In fact, the lack of a congruency effect for the older participants suggests that they are better able to suppress the distractors than younger participants are, which in turn may have led to the improvement in accuracy.

When the flanker stimuli were presented for 200 ms or 100 ms, the younger participants exhibited a standard white-bear effect, but the older participants appear to show an attenuated white-bear effect. Although neither of the Age × Flanker configurations reached the 0.05 significance level, the simple main effects analysis clearly showed a difference in performance. Further, for the 100-ms condition, the Bayes factor analysis provided some additional support that there was an age-related difference in performance. Notably, the difference in performance appears to be larger in the 100-ms condition, ostensibly due to it being a more difficult condition. Overall, these data suggest that older participants were not effectively proactively allocating attention to the flankers, whereas younger participants were. In both the 200-ms and 100-ms conditions, younger participants exhibited a congruency effect during the flanker task, but older participants did not. We further investigated this lack of a congruency effect and white bear by looking at the relationship between the two in the 100-ms condition (since the difference in the white bear was more pronounced). Crucially, the correlation analysis suggested that the white-bear effect was actually associated with increased distractor interference for the older participants, but not for the younger participants. Although, given the small sample size, this analysis is exploratory at best.

Furthermore, in both the 200-ms and 100-ms experiments, the low-contrast condition did not induce a congruency effect, whereas the equal-contrast condition did, suggesting that the flankers in the low-contrast condition did not require significant attentional resources to process and inhibit. This is consistent with prior literature showing that dimmer flankers lead to reduced conflict cost (Wyatt & Machado, 2013; Zeischka, Coomans, Deroost, Vandenbossche, & Soetens, 2011, Experiment 2).

## General discussion and conclusions

In the present study, we aimed to assess proactive allocation of attention to expected distractor location in old age. While previous studies have pointed to impaired proactive control in old age, such studies focused almost solely on measures of inhibition rather than allocation. To test this issue, we used the attentional-white-bear paradigm (AWB; Tsal & Makovski, 2006), which provides a direct measure of the proactive allocation of attention to expected distractor locations. In Experiment 1, we provided a direct replication of Tsal and Makovski (2006), in both younger and older participants. Both groups demonstrated intact proactive allocation of attention to the expected distractor location, and to the same degree. However, in Experiment 1 participants had an unlimited amount of time to see the stimuli. In Experiments 2a and 2b, we taxed the attentional system more by decreasing the flanker stimulus presentation time. Under these conditions, an age-related deficit in the allocation of attention toward expected distractors emerged. In particular, older participants showed reduced allocation of attention compared with their younger counterparts. A secondary finding was that this reduced allocation of attention during the temporal-order-judgment task was associated with better inhibition during the flanker task, but only for the older participants.

Although the proactive allocation of attention elicited by the AWB yields a seemingly benign effect in younger controls, that does not mean it is not the same mechanism engaged by other processes (albeit used to a different end). This is crucial because our main finding is that allocation of attention was reduced in our older population when the attention system was taxed sufficiently. Therefore, allocation of attention may be impaired in other processes as well, such as visual marking. Studies of visual marking and aging have shown no age-related differences in performance in static displays (Kramer & Atchley, 2000; Kramer & Kray, 2006), but, notably, they have emerged in dynamic displays (Becic et al., 2007; Watson & Maylor, 2002). This may be attributed to the fact that dynamic displays are more difficult than static displays, similar to how differences in the AWB did not emerge until the stimulus presentation time was reduced. It has been shown that, in visual marking studies, the inhibition in the preview search acts via a location-based inhibitory bias against the to-be-ignored distractors (Humphreys et al., 2004; Olivers et al., 2006; Watson & Humphreys, 2004). Therefore, since a moving target (i.e., a dynamic display) may only be in a location for a limited amount of time, this may effectively act as a short stimulus presentation time at each given location (inducing age-related differences like in our study). This is speculative, of course, but future research could assess these possibilities.

A secondary finding was that, for the older participants (during equal-contrast trials), the magnitude of the AWB effect during the temporal-order-judgment task correlated with the magnitude of the congruency effect during the flanker task. In other words, more proactive allocation of attention in the temporal-order-judgment task seems to be associated with worse inhibition in the flanker task. This makes sense, because if older participants do not allocate their attention to the expected location of the distractor (low white bear), then they may never process them in the first place, leading to a lower or eliminated congruency effect because there is nothing to inhibit. An alternative possibility is that this may be attributed to impaired attentional disengagement (Greenwood & Parasuraman, 1994; Owsley, 2016) in older populations. Older participants who allocated their attention toward the expected distractor locations more often or efficiently may not have been as able to disengage from the distractor, leading to enhanced processing of the flankers and larger congruency effects. This is in line with recent findings from our lab where older participants were less likely to rapidly reject a salient nontarget compared to younger participants (Ashinoff, Geng, & Mevorach, 2019). Given that the white-bear effect was reduced in older populations relative to younger populations, it seems more likely that this effect is being driven by reduced distractor processing. Somewhat surprisingly, for the younger participants there was no relationship between the allocation of attention to expected flanker locations during the temporal-order-judgment task and inhibition of those distractors during the flanker task. This has implications for any future interpretations of the AWB effect in healthy, younger controls, as it now seems unlikely that the AWB plays a beneficial, proactive role in inhibition processes. Furthermore, if the allocation of attention in the white-bear paradigm is not a benefit to subsequent inhibition processes, then another possibility is that the age-related reduction in the white bear is a strategic choice, perhaps a result of a rapid disengagement impairment.

It is important to note that the attentional-white-bear paradigm and its eponymous effect has never been defined by inhibition taking place following allocation. In fact, the task design guarantees that allocation cannot be followed directly by inhibition (the temporal-order-judgment task and the flanker task occur on independent trials). In previous studies (Tsal & Makovski, 2006), and in ours, it was inferred that attention was being proactively allocated toward the expected distractor location in anticipation of having to subsequently inhibit those locations, but this has always been an assumption and has not been directly tested. Even our result must be interpreted with care since it only indirectly measures this potential relationship and has a small sample size for a correlational analysis.

In summary, older participants do exhibit an age-related difference in performance during the AWB when the attentional system is sufficiently taxed, reflecting differences in the allocation of attention to the location of an expected distractor. Future research will have to establish if this difference reflects an impairment or a strategic choice, since the allocation of attention seems to convey no obvious benefit in this paradigm.

### Authors note

The authors would like to thank Denise Clissett for her instrumental role in recruiting older participants, as well as Samantha Ellis, Charlie-Louise Milward, Alice Sarhanis, and Leah Supra for their contributions to this project as undergraduate research assistants. This paper is based on Chapter 5 of author B.K.A’s dissertation (Ashinoff, 2017). This research was funded in part by a grant from the ISF (Grant No. 1898/2015) awarded to Yehoshua Tsal. Brandon K. Ashinoff is currently supported by a T32 Postdoctoral Fellowship (T32-MH018870) at Columbia University. Experimental data from this study and program code are publicly available via the Open Science Framework (OSF): https://osf.io/f576c/
